# Pulmonary Arterial Hypertension: Emerging Principles of Precision Medicine across Basic Science to Clinical Practice

**DOI:** 10.31083/j.rcm2311378

**Published:** 2022-11-09

**Authors:** Neil J. Kelly, Stephen Y. Chan

**Affiliations:** 1 Center for Pulmonary Vascular Biology and Medicine and Pittsburgh Heart, Lung, and Blood Vascular Medicine Institute; Division of Cardiology; Department of Medicine, University of Pittsburgh School of Medicine and University of Pittsburgh Medical Center, Pittsburgh, PA 15213, USA

**Keywords:** pulmonary hypertension, disease mechanism, systems biology, translational biology, endothelium, precision medicine

## Abstract

Pulmonary arterial hypertension (PAH) is an enigmatic and deadly vascular disease with no known cure. Recent years have seen rapid advances in our understanding of the molecular underpinnings of PAH, with an expanding knowledge of the molecular, cellular, and systems-level drivers of disease that are being translated into novel therapeutic modalities. Simultaneous advances in clinical technology have led to a growing list of tools with potential application to diagnosis and phenotyping. Guided by fundamental biology, these developments hold the potential to usher in a new era of personalized medicine in PAH with broad implications for patient management and great promise for improved outcomes.

## 1. Introduction

Pulmonary hypertension (PH) is a complex and progressive disease involving elevated pressures in the pulmonary arteries due to one or multiple varied etiologies. Left untreated, PH is associated with right ventricular (RV) hypertrophy and failure resulting in markedly reduced life expectancy. Insights and mechanistic discoveries over the past two decades have begun to untangle this enigmatic and, so far, incurable disease. As a result, novel approaches to PH management are emerging which set the stage for introducing an era of precision medicine—holding the potential to bring earlier diagnoses, more effective treatments, and improved patient outcomes. This review will discuss key principles of the scientific basis of PH and its clinical management while highlighting emerging and potentially practice-changing concepts and technologies.

## 2. Clinical Definitions

Prior to our current understanding of disease mechanisms, PH was classified as either “primary” (idiopathic) or “secondary” to any of a variety of diverse clinical states. In 1998, a working group of the World Society of Pulmonary Hypertension (WSPH), sponsored by the World Health Organization (WHO), devised the current schema of clinical groupings which aims to categorize PH according to its etiology [1]. In the most recent iteration drafted by the 6th WSPH [2], PH is defined by a resting mean pulmonary artery pressure (mPAP) greater than 20 mmHg by right heart catheterization and categorized according to additional hemodynamic and clinical factors: Pulmonary arterial hypertension (PAH – WSPH Group 1), PH secondary to left heart disease (PH-LHD – WSPH Group 2), PH secondary to chronic lung disease or hypoxia (PH-CLD – WSPH Group 3), chronic thromboembolic pulmonary hypertension (CTEPH – WSPH Group 4), and PH due to multifactorial or miscellaneous causes (WSPH Group 5). To be classified as WSPH Group 1 PAH, precapillary hemodynamics must be observed, with mPAP >20 mmHg accompanied by an elevated pulmonary vascular resistance (PVR) of greater than or equal to 3 Wood units (mmHg/L/min) and pulmonary artery wedge pressure (PAWP) less than or equal to 15 mmHg. Importantly, expert clinical assessment must rule out predominant contributions by left heart disease, hypoxic lung disease, and chronic pulmonary emboli. On the other hand, PH secondary to left heart disease (PH-LHD – WSPH Group 2) is defined by a PAWP greater than 15 mmHg regardless of PVR. In clinical practice, patients often fall into more than one category of PH [2].

While epidemiological data comparing across PH groups are less available and often limited to diagnoses inferred from echocardiography, it is clear that PAH constitutes a minority of total global PH burden, but the exact prevalence of PAH is not known. In total, PAH is diagnosed in an estimated 2.4–7.6 million individuals annually with a prevalence of 15 to 50 million cases and a strong female predominance [3,4]. This may differ, depending upon geography or epidemiologic techniques. For example, in a large population-based cohort study from Ontario, Canada, the annual prevalence of any form of PH was 127.3/100,000 of which PAH accounted for 15.6% [5], while an Australian cohort identified the proportion of overall PH prevalence due to PAH at 2.7% [6]. In recent years, the foundational and clinical understanding of PAH has advanced dramatically, and this review will focus on recent progress made in the field of PAH and possible clinical advancements in the near future.

## 3. Etiology

PAH describes a clinical syndrome in which primary remodeling of the small muscularized arterioles and precapillary vessels (50–500 *μ*m diameter) promotes pathologic increases in pulmonary vascular resistance, culminating in elevated pulmonary artery pressures, RV hypertrophy, and symptomatology and death from RV failure [7]. A minority of PAH cases (2–3%) can be directly attributed to heritable causes (HPAH) [8], while roughly 50% of PAH cases are classified as idiopathic (IPAH). However, it is increasingly recognized that a substantial proportion—20–30%—of cases labeled as idiopathic are likely to be hereditary [9]. Much of the remaining PAH burden is derived from connective tissue diseases (systemic sclerosis, lupus, rheumatoid arthritis, and others). Rare causes of PAH have been linked to associated triggers including portopulmonary hypertension, drugs/toxins (with recent increasing cases of methamphetamine use), infections (human immunodeficiency virus [HIV], schistosomiasis), congenital heart disease, pulmonary venoocclusive disease (PVOD)/pulmonary capillary hemangiomatosis (PCH), and persistent pulmonary hypertension of the newborn (PPHN) [2].

With the exceptions of the rare entities of PVOD/PCH [10] and PPHN [11], the various causes of PAH share similar histopathological features. PAH is characterized by resistive changes to the small precapillary arterioles including medial hypertrophy and hyperplasia, intimal and adventitial fibrosis, and thrombotic and plexiform lesions [12]. Over variable time frames, the progression and accrual of vascular remodeling manifests as clinical disease.

## 4. Disease Mechanisms (Fig. 1)

### 4.1 Disrupted Homeostasis of Vascular Effectors

Prior to our current understanding of genetic disease influences, it was apparent that the vasoconstrictive phenotype of PAH was provoked in part by endothelial dysfunction and disrupted homeostasis between various mediators of vascular tone. Among the best studied are the vasodilatory arachidonic acid metabolite prostacyclin and free radical nitric oxide (NO), as well as the vasoconstrictive peptide hormone endothelin-1 (ET-1, also known as EDN1); manipulation of these vasoactive mediators forms the basis of current pharmacotherapy in PAH [13].

Prostacyclin (PGI2) is a potent vasodilator and inhibitor of platelet activation derived from the arachidonic acid metabolite prostaglandin H2 (PGH2) [14]. Examination of the urine of PAH patients has shown that PGI2 breakdown products are decreased while those of the vasoconstrictive and platelet-activating PGH2 derivate thromboxane A2 (TXA2) are increased [15]. Additionally, prostacyclin synthase, which catalyzes the conversion of PGH2 to PGI2, is decreased in the lungs of IPAH patients [16], favoring increased flux of PGH2 towards TXA2.

NO is synthesized from L-arginine through the actions of the nitric oxide synthase (NOS) isoenzymes in concert with multiple cofactors [17]. Among its many effects, NO generally causes vasodilation while inhibiting pulmonary artery endothelial cell (PAEC) apoptosis, PA smooth muscle cell (PASMC) proliferation, and platelet aggregation—all key pathologic features of PAH. Numerous mechanisms contribute to a reduction in bioavailable NO in the setting of PAH, including decreased NOS expression, decreased substrate availability by upregulation of arginases, cofactor oxidation, and rapid scavenging by local reactive oxygen species (ROS) (reviewed in [18]). While endothelial NOS (NOS3, also known as eNOS) expression is decreased in lung sections from PAH patients [19], it is paradoxically increased in plexiform lesions [20]; however, eNOS is unlikely to contribute significantly to NO synthesis in this environment where it probably exists in an uncoupled state and catalyzes the formation of the superoxide radical, promoting oxidative stress and pulmonary vascular remodeling [21].

ET-1 is a peptide hormone primarily synthesized in the endothelium where it is translated as a prepropeptide and undergoes two stages of proteolytic activation to reach its mature form. ET-1 exerts its effect through the actions of two G-protein coupled receptors, ET-A and ET-B, localized on the smooth muscle cells. Expression of ET-1—as well as its associated activating proteases and receptors—is increased in PAH [22], where it directs a program of vasoconstriction and PASMC proliferation (reviewed in [23]).

### 4.2 Genetics

Over two decades ago, the discovery of causative heterozygous bone morphogenetic protein 2 (*BMPR2*) mutations within familial cases of PAH [24,25] marked a foundational moment in our understanding of the disease. *BMPR2* encodes a membrane-bound type 2 receptor of the transforming growth factor beta (TGF*β*) receptor superfamily which heterodimerizes with type 1 receptors and, upon ligand binding, canonically transduces cytosolic and transcriptional signals through the “mothers against decapentaplegic” SMAD1/5/9 signaling pathway [26]. It is estimated that *BMPR2* mutations account for roughly 75% of HPAH and perhaps as much as 25% of IPAH [27]. Inherited in an autosomal dominant pattern, the penetrance of HPAH due to *BMPR2* mutations is estimated to be 14% in males and 42% in females [28], indicating that sex and other factors play a strong role in disease manifestation.

Mutations in other BMPR2-related genes have been linked to PAH albeit at lower frequencies, including type I receptors (*ACVRL1*, *ENG*), SMAD family members (*SMAD4*, *SMAD9*), and BMPR2 ligands (*GDF2* which encodes BMP9) [29–33]. Pathogenic *BMPR2* mutations result in BMPR2 haploinsufficiency with a decrease in pulmonary vascular BMPR2 protein expression; notably, BMPR2 expression is also decreased in the pulmonary vasculature of patients with other forms of pulmonary hypertension, suggesting mechanistic parallels between the various subgroups [34]. More recently, rare PAH-associated mutations have been identified in genes and/or neighboring chromosomal loci without a clear link to BMPR2 signaling, including caveolin 1 (*CAV1*) [35], potassium channel subfamily K, member 3 (*KCNK3*), probable cation-transporting ATPase 13A3 (*ATP13A3*) [36], aquaporin 1 (*AQP1*) [36], SRY-box 17 (*SOX17*) [36,37], and major histocompatibility complex, class II, DP alpha 1 and beta 1 (*HLA-DPA1/HLA-DPB1*) [37]. It is hoped that greater clarity into the functional consequences of these rare disease-associated mutations will bring a fuller picture of the mechanistic underpinnings of PAH.

### 4.3 BMPR2 Insufficiency

The exact molecular mechanisms that explain the link of BMPR2 haploinsufficiency to vascular remodeling and PAH remain incompletely defined; however, significant progress has been achieved in recent years in understanding this complex paradigm. *In vitro* studies have demonstrated that under normal circumstances, ligand binding to BMPR2 protects PAECs from apoptosis [38] while suppressing the proliferation of PASMCs [39,40]. Phenotypically, BMPR2-deficient pulmonary artery endothelial cells display increased apoptosis [38], disrupted vasodilator/vasoconstrictor homeostasis [41], endothelial-to-mesenchymal transition (EndoMT) [42], and dysregulated metabolism [43–45]—alterations which mirror those seen in cultured PAECs from PAH patients in general. In models of BMPR2 insufficiency, rodents with heterozygous knockout of *Bmpr2* are phenotypically similar to their wild-type counterparts at baseline but develop PAH under inflammatory stress [46,47]. In this respect, animal models suggest the requirement for a second hit as an explanation for the low penetrance of PAH in BMPR2 haploinsufficiency. In PAH subjects, disruption of BMPR2 signaling and the downstream SMAD1/5/9 cascade is accompanied by increased pathologic TGF*β* signaling through SMAD2/3 [48]. Recently, Yung and colleagues [49] found that the activin and growth and differentiation factor (GDF) ligands activin A, GDF8 and GDF11 were upregulated in the lungs of PAH subjects and activated a SMAD2/3-mediated proproliferative, antiapoptotic phenotype in PAECs and PASMCs. When these effectors were antagonized with a ligand trap, SMAD2/3 signaling was attenuated and experimental PH was reversed, suggesting restoration of balance to amongst BMP versus TGF*β*/Activin/GDF as a therapeutic target in PAH. In addition to canonical SMAD signaling, BMPR2 effector functions may vary depending on context and the engagement of noncanonical pathways (reviewed in [50]) and cell types beyond endothelium and smooth muscle cells, adding further complexity to its pleiotropic roles.

### 4.4 Acquired & Environmental Factors

Data from the Registry to Evaluate Early and Long-Term Pulmonary Arterial Hypertension Disease Management (REVEAL) suggest that drugs and toxins are responsible for approximately 1 in 20 total PAH cases [8]. Among the earliest drugs associated with PAH was the anorexigen aminorex (2-amino-5-phenyl-2-oxazoline) [51], whose association with an epidemic of PAH in the 1960s led to heightened awareness of PAH in general [52]. Since then, additional stimulant anorexigens—notably fenfluramine/dexfenfluramine [53,54]—as well as recreational amphetamines and other drugs have been linked to the development of PAH [2,55]. The known serotonergic properties of these toxins sparked interest in serotonin (5-hydroxytryptamine, 5HT) as a molecular mediator of disease [56]. 5HT promotes smooth muscle cell proliferation and pulmonary vasoconstriction [57,58], and the active metabolite of dexfenfluramine is a potent activator of the 5HT2B receptor [59]. Additionally, genetic deletion or pharmacologic blockade of 5HT1B [60] or 5HT2B [59] is protective against hypoxia-induced rodent PH. In human IPAH, plasma 5HT is increased [61] suggesting a broader role for 5HT beyond anorexigen-induced PAH. Interestingly, 5HT exacerbates hypoxia-induced PH in BMPR2-deficient mice while inhibiting BMP signaling via SMAD proteins, suggesting mechanistic overlap with HPAH [62].

The cellular response to hypoxia has long been recognized to play a crucial role in the pathogenesis of PAH. While chronic exposure to hypoxia and high altitude retains a separate designation within Group 3 PH, hypoxic signaling pathways are intimately involved in PAH and other WHO groups [63]. In the pulmonary vasculature, acute hypoxia leads to vasoconstriction through transcriptional reprogramming to promote the synthesis of vasoconstrictors such as ET-1 [64] over vasodilators including NO [65]; with sustained hypoxia, remodeling leads to alterations in pulmonary vascular architecture and pulmonary hypertension [66]. The hypoxia inducible factors (HIFs) are master transcriptional regulators of hypoxic cellular responses composed of an oxygen-sensitive alpha subunit (HIF1*α*, HIF2*α*, HIF3*α*) and an oxygen-insensitive beta subunit (aryl hydrocarbon receptor nuclear translocator ARNT1, ARNT2, ARNT3) shared with the aryl hydrocarbon receptor (AhR) [67]. In normoxic conditions, the alpha subunit is marked for ubiquitination and degradation by prolyl hydroxylase domain (PHD) mediated hydroxylation leading to binding of the von Hippel Lindau (VHL) E3 ubiquitin ligase complex and subsequent degradation. In hypoxic conditions, the alpha subunit heterodimerizes with the beta subunit and translocates to the nucleus where it initiates transcriptional events through binding to HIF response elements (HREs). It is increasingly appreciated that HIF complexes can be stabilized by alternative means in normoxic conditions, including the conditions of inflammation, mechanical stretch, and metabolic stress characteristic of PAH (reviewed in [63]). Additionally, a variant in the *EPAS1* gene encoding HIF2*α* has been associated with the development of RV failure in cattle living at high altitude, known as Brisket Disease, adding evidence of genetic influences on aberrant HIF activation in PH [68].

More recently, it has been demonstrated by independent laboratories that the aryl hydrocarbon receptor (AhR), a master regulator of xenobiotic responses which shares a heterodimerization partner with the HIF*α* subunits, is of critical importance in experimental PH [69,70]. In rodent models of PH, the phenotype triggered by Sugen (SU5416) as a model of PAH was attributed to its activation of AhR rather than inhibition of VEGFR2 as previously accepted [71]. AhR activation provoked inflammation and PH in animal models, while plasma agonistic AhR activity was higher in PAH patients than in healthy controls [70]. If this finding is upheld, it would suggest a role for countless additional environmental xenobiotics in the development of PAH. For example, a recent epidemiological study based in the United Kingdom found that air pollution may be linked to PAH outcomes [72], although the mechanism of this association has not yet been explored.

### 4.5 Sex Differences

The female predominance of PAH is well-established and extends to nearly all subgroups of the disease [4,8]. While females outnumber males by more than 2:1 in national registries [3], prevalent males have a significantly higher mortality. This observation that females have better outcomes in the context of higher disease burden has been termed the “estrogen paradox” [73]; these findings have spurred investigation of the effects of sex hormones—predominantly the major female estrogen, estradiol (17*β*-estradiol, E2)—and their metabolites in PAH. As with humans, model organisms also exhibit sexual dimorphism whereby males experience more severe disease than females [74,75], while exogenous estradiol administration prevents and reverses rodent PH [76,77]. Estradiol modulates transcriptional programming through binding to alpha (ER*α*, also known as nuclear receptor subfamily 3, group A, member 2 [NR3A1]) or beta (ER*β*, also known as NR3A2) hormone receptors as well as non-genomic effects on binding to G-protein coupled estrogen receptors [78]. In pulmonary vascular cells, estradiol generally yields an anti-mitogenic, anti-proliferative phenotype in PASMCs while inducing the synthesis of vasodilatory mediators [73].

However, the actions of estradiol are complex and context-dependent across its metabolites and target cell types. The first step in estradiol metabolism is mediated by the cytochrome P450 (CYP450) superfamily of heme-containing monooxygenases and predominantly involves hydroxylation at the C2 and C4 positions, although hydroxylation at other carbons including C16 also occurs [79]. Broadly speaking, the anti-mitogenic, anti-proliferative 2-estrogens are thought to be protective in PAH, while the pro-inflammatory, pro-proliferative 16-estrogens are believed to be pathogenic (reviewed in [73]). CYP1B1, which hydroxylates estrogen at the C4 and, to a lesser degree, C2 and C16 positions, is of particular interest as it is highly ex pressed in PASMCs isolated from PAH patients [80], and genetic polymorphisms affecting CYP1B1 protein function have been associated with decreased ratios of “good” 2-estrogens metabolite to “bad” 16-estrogen metabolites as well as PAH penetrance in females with *BMPR2* mutations [81]. In rodent models of PAH secondary to serotonergic excess—including after administration of the anorexigen dexfenfluramine implicated in drug-induced PAH—CYP1B1 and 16-estrogen levels are increased, while genetic knockout or pharmacologic inhibition of CYP1B1 prevents disease [82,83]. CYP1A1, meanwhile, is also involved in estrogen metabolism and is upregulated in experimental PAH through activation of AhR, although further work is needed to define its relationship to estrogen signaling in PAH [69].

Upstream of its metabolism, estradiol is synthesized from androgens through the action of aromatase (CYP19A1) [79]. Implicating a pathogenic role for estrogens, a polymorphism in *CYP19A1* was associated with increased estradiol levels and the presence of portopulmonary hypertension (PoPH)—a subgroup of PAH—in patients with advanced liver disease, while PoPH was also associated with increased levels of 16-estrogens [84]. Interestingly, roughly one-third of PAH patients in the US-based REVEAL registry were classified as obese at the time of enrollment [85], and visceral adipose tissue is known to be a major site of aromatase expression and estrogen biosynthesis [86]. In leptin-deficient obese mice, which spontaneously develop PH and pulmonary vascular remodeling, these pathophenotypes were attenuated by aromatase inhibition with anastrozole or CYP1B1 inhibition with 2,2’,4,6’-tetramethoxystilbene, suggesting a mechanistic role for estrogen in this disease model [87]. In a distinct rodent model of PH, the anti-diabetic drug metformin was shown to reverse PH and vascular remodeling through transcriptional repression of aromatase, again suggesting a therapeutic effect of estrogen inhibition [88]. In a small randomized trial comparing anastrozole to placebo in PAH patients on background therapy, anastrozole was associated with a modest improvement in 6-minute walk distance after 3 months while having no effect on an echocardiographic metric of RV systolic function [89]. Taken together, these findings indicate important influences including estrogen metabolism, cell type, and disease model in defining the effects of estrogens on pulmonary vascular remodeling.

In contrast to its variable effects on the pulmonary vasculature, the beneficial effects of estradiol on RV structure and function are well-established and thought to explain in large part the estrogen paradox [90]. In human cohorts, the estrogen-diminished state of menopause has been associated with onset of CTD-PAH [91], while post-menopausal women receiving hormone replacement therapy have evidence of improved RV systolic function on cardiac imaging [92]. Meanwhile, females have evidence of improved RV adaptation to PAH by invasive [93] and non-invasive [94] methods as compared to males. In animal models of PH, females develop less severe RV hypertrophy (RVH) [74], while estradiol administration attenuates RV remodeling [76,95,96]. Historical studies have demonstrated that estradiol inhibits cardiac fibrosis in models of left ventricular (LV) failure via an ER*β*-dependent mechanism [97]. Recently, however, experimental models have shown a critical role for ER*α* in orchestrating adaptive RV remodeling, at least in part through a BMPR2-dependent mechanism [98,99]. The success of pharmacologic manipulation of estrogen signaling in PAH will likely depend on the ability to balance its opposing effects which define the estrogen paradox.

There remain significant gaps in our understanding of PAH during the unique state of pregnancy. Although estrogen levels increase throughout pregnancy [100], PAH in pregnancy poses serious maternal and fetal risks (reviewed in [101]) such that current guidelines recommend against conception [102]. These risks are thought to be mediated by hemodynamic and hemostatic changes of pregnancy, yet the precise molecular mediators and roles of sex hormones are understudied and may hold novel insights into the effects of pregnancy-specific estrogen derivatives.

Finally, in contrast to the pleiotropic effects of female sex hormones, recent evidence has indicated a protective effect of the Y chromosome in experimental hypoxic PH [103,104]. A subsequent study found that the transcription factor SRY, encoded on the Y chromosome, is a direct positive regulator of *BMPR2* expression at the transcriptional level [105], providing an additional mechanistic explanation for sex disparities in PAH.

### 4.6 Dysregulated Metabolism

The transition from oxidative phosphorylation to aerobic glycolysis in normoxic conditions, known as the Warburg effect (named the Pasteur effect in hypoxia), is a hallmark of PAH [106]. Central to this shift is the inhibition of pyruvate dehydrogenase, which catalyzes conversion of pyruvate to acetyl-CoA allowing progression from glycolysis to the tricarboxylic acid (TCA) cycle. In PAH-relevant pathways including hypoxia and tyrosine kinase signaling [107,108], this intermediate step is blocked via upregulation of the inhibitory enzyme pyruvate dehydrogenase kinase (PDK) thereby shunting pyruvate to glycolysis. In experimental PH, the PDK inhibitor dichloroacetate (DCA) prevents and reverses pulmonary hypertension and causes apoptosis of PASMCs [109–111]. Interestingly, an open-label study of DCA in IPAH patients on baseline therapy led to variable reductions in mean PA pressure, with lack of response predicted by genotypic variants in key mitochondrial genes [112]. Additional factors, including inflammation and BMPR2 deficiency, have also been suggested to contribute to the Warburg effect in PAH. Recent studies have also shown that anaerobic glycolysis is favored in PAH by alternative splicing of pyruvate kinase muscle (PKM) isoforms in response to downregulation of microRNA-124 (miR-124), a process linked to BMPR2 deficiency [44,113]. Additional PAH-related mediators, including the inflammatory cytokine TNF-*α* [114] as well as HIV infection [115] have been proposed to contribute to Warburg physiology, demonstrating the overlap between various pathophysio-logic influences in PAH.

As seen in the Warburg effect, the metabolic shift to anaerobic glycolysis facilitates the use of cellular carbons to generate biomass and meet the anabolic demands of rapid proliferation [116]. In a process known as anaplerosis, the TCA cycle intermediate oxaloacetate is replenished to maintain the pool of biosynthetic and bioenergetic precursors either through the actions of pyruvate carboxylase on pyruvate or deamidation of glutamine (“glutaminolysis”) by glutaminase [117]. In experimental PAH, glutaminase (GLS1) is upregulated in a yes-associated protein (YAP1)-dependent fashion in order to generate macromolecular precursors and sustain proliferation, with inhibitors of these proteins leading to prevention and reversal of rodent PH [118–120]. Interestingly, NO has been shown to promote Warburg-type physiology and glutaminolysis in ovarian cancer cells, although it is unknown whether comparable mechanisms translate to pulmonary vascular cell types [121].

Electron transport is a critical feature of mitochondrial metabolism and requires the presence of evolutionarily ancient iron-sulfur (Fe-S) clusters, bioinorganic prosthetic groups which facilitate cellular redox processes. Fe-S cluster biogenesis requires more than 30 cytosolic and mitochondrial proteins, and synthetic dysfunction attenuates oxidative phosphorylation as well as other critical metabolic and cellular events including DNA repair [122, 123]. Our group previously identified a hypoxia-inducible microRNA, microRNA-210 (miR-210), which translationally represses the Fe-S cluster assembly enzymes ISCU1/2 and is upregulated in PAH [124,125]. Importantly, both forced overexpression of miR-210 or pharmacologic silencing of *ISCU* promoted experimental PAH [124]. Mutations in Fe-S biogenesis proteins are also associated with several Mendelian disorders, including Friedreich’s Ataxia (Frataxin, *FXN*) and the multiple mitochondrial dysfunction syndromes (MMDS) 1 (NFU1 iron-sulfur cluster scaffold, *NFU1*) and 2 (BolA family member 3, *BOLA3*) [126, 127]. PAH is frequently associated with the clinical syndrome of MMDS1, and rats harboring a human *NFU1* mutation develop spontaneous PH [128–130]. Additionally, we have shown that deficiency in BOLA3 or FXN causes experimental PH through multiple mechanisms related to Fe-S biology including attenuation of oxidative phosphorylation, accumulation metabolic intermediates, and induction of cellular senescence [131,132]. Collectively, these findings indicate a critical role for Fe-S clusters in the maintenance of metabolic integrity and normal cellular proliferation—processes which, when perturbed, contribute to pulmonary vascular remodeling.

It is increasingly appreciated that pathologic metabolic abnormalities in PAH extend well beyond mitochondrial flux. Recent evidence has pointed to metabolic dysfunction and aberrant insulin signaling and lipid handling in multiple forms of PH, including PAH [133] and with BMPR2 deficiency [134]. Our group and others have shown a critical role for the nuclear receptor peroxisome proliferator-activated receptor gamma (PPAR*γ*), a master regulator of glucose and fatty acid utilization, in PAH pathogenesis [119,135,136]. Upregulation of the microRNA miR-130/301 in PH family led to translational repression of PPAR*γ* with cell-type and context-dependent effects on PAEC and PASMC proliferation resulting in pathologic vascular remodeling [119]. PPAR*γ* has also been identified as a downstream mediator of non-canonical SMAD1/5/9-independent BMP2/BMPR2 signaling where it functions to repress PASMC proliferation [137]. The protective actions of PPAR*γ* extend to the failing right ventricle, where it exerts therapeutic effects by restoring homeostasis to glucose utilization and fatty acid oxidation [136]. In sum, the diverse and growing list of metabolic perturbations in PAH is reflective of the multidimensional links between metabolism and pulmonary vascular homeostasis.

### 4.7 Inflammation & Immune Activation

Perivascular inflammation involving macrophages, dendritic cells, T and B lymphocytes, and mast cells is a characteristic feature PAH and correlates with vascular remodeling [138,139], suggesting a causal relationship between the two. Myeloid cell recruitment from bone marrow and blood has been highlighted as a key process in the development of vascular inflammation in PAH [140]. Furthermore, targeting right ventricular inflammation via the NLRP3 inflammasome has recently been described [141]. Correspondingly, circulating cytokine levels, including interleukin-1*β* (IL-1*β*), interleukin-6 (IL-6), and tumor necrosis factor-*α* (TNF-*α*) are increased in PAH and correlate with mortality [142–144]. In mouse models, transgenic overexpression of the proinflammatory cytokine interleukin-6 (IL-6) is sufficient to cause PH, while its deficiency exerts protective effects against disease development [145]. Interestingly, cultured PASMCs treated with silencing RNA to BMPR2 overexpress IL-6, and IL-6 is upregulated in mice harboring a dominant negative *Bmpr2* transgene [146], thus linking proinflammatory cytokine expression to BMPR2 deficiency. Inflammatory cytokines can directly induce PAH-relevant phenotypes including proliferation in cultured PASMCs via induction of mitogenic stimuli [147,148]. Moreover, pharmacologic antagonism of IL-1*β* and IL-6 receptors is protective in experimental PH [149,150], although clinical trials of IL-6 receptor blockade in PAH have questioned its translatability [151]. Taken together, these points underscore a complex and unresolved interplay between inflammatory mediators and PAH.

The CTD-PAH subgroup is characterized by systemic immune dysregulation and autoimmunity. Studies of T lymphocyte populations in PAH have suggested that regulatory T cells play an integral role in the maintenance of vascular integrity and protect against the development of PAH [152]. Autoantibodies to endothelial cell antigens have been described in scleroderma-associated PAH and induce apoptosis in cell culture [153], although further study of the exact role of anti-endothelial antibodies and B cell depletion in CTD-PAH requires further study [55].

PAH secondary to infection is also thought to be at least partly related to particular inflammatory signatures. While incompletely understood, the pathogenesis of HIV-PAH is likely multifactorial with contributions from the direct actions of viral proteins, inflammatory mediators, and other factors leading to pulmonary vascular remodeling (reviewed in [154]). Expanding on this concept, Saito and colleagues [155] offered recent evidence that endogenous human retroviruses contribute to PAH pathogenesis, as well. In their study, they showed that transcripts of human endogenous retrovirus K (HERV-K) are upregulated in the lungs of PAH patients and that HERV-K proteins can drive pathogenic vascular changes in rodent models of PH, suggesting that both exogenous and endogenous viruses can modulate inflammatory signatures to promote PAH. Recently, observational data showed that, while the incidence of Coronavirus Disease 2019 (COVID19) in PAH is similar to that of the general population, outcomes are significantly worse [156,157]. There is currently insufficient evidence to suggest a mechanistic link between the causative severe acute respiratory syndrome coronavirus 2 (SARS-CoV-2) infection and PAH, and the poor outcomes may result, at least in part, from the inherent fragility of the PAH patient population.

Schistosomiasis-associated PAH (Sch-PAH), caused by infection with the helminth *Schistosoma mansoni*, is a common cause of PAH globally, though its mechanisms remain ill-defined (reviewed in [158]). In murine models of Sch-PAH, codeletion of the T_H_2 cytokines IL-4 and IL-13 protects against the development of experimental PH, which is thought to be related to IL-13-mediated upregulation of TGFb with consequent SMAD2/3 activation and PASMC proliferation [159,160]. Interestingly, IL-13 overexpression induces experiment rodent PH [161], and plasma IL-13 is elevated in scleroderma-associated PAH as compared to scleroderma without PAH [162], suggesting a plausible role for type 2 inflammation in PAH more generally.

As evidenced by the myriad inflammatory mediators associated with PAH, their precise role in disease progression is complex and incompletely understood (reviewed in [163]). Recently, the application of high-throughput techniques has been helpful in defining PAH-associated inflammatory and immune signatures [164,165], and longitudinal studies will add additional clarity given the dynamic nature of tissue inflammation [166].

### 4.8 Epigenetics

Epigenetic modifications describe heritable changes in gene expression that do not alter DNA sequence and mainly comprise DNA methylation, histone modifications, and changes in non-coding RNA expression [167]. As alluded to above, non-coding RNAs (ncRNAs) have been implicated in multiple pathways related to PAH pathogenesis. ncRNAs fall into several categories, including microRNAs (miRNAs), long non-coding RNAs (lncRNAs), and many others [168] where contributions to PAH are only beginning to be appreciated. miRNAs exert their effects through sequence-dependent binding and posttranscriptional repression of target mRNAs in order to orchestrate the downregulation of a wide range of targets [169]. The importance of miRNAs in PAH is well-established, as they have been shown to affect a number of PAH-relevant pathways related to BMPR2, hypoxia, estrogen, PPAR*γ*, inflammation, and more (reviewed in [170]). lncRNAs are single-stranded RNAs with multiple potential functions, most notably as facilitators of chromatin modification, although additional roles in miRNA antagonism and scaffolding have been suggested [171]. Recent discoveries have shed light onto the involvement of lncRNAs in PAH pathobiology; notably, tyrosine kinase receptor–inducing lncRNA (TYKRIL) was recently shown to be upregulated in PASMCs and pericytes from PAH patients and promotes cellular proliferation by interfering with p53-mediated transcriptional repression of platelet-derived growth factor receptor beta (PDGFR*β*) [172]. Given the abundant and growing understanding of ncRNAs in PAH pathology, it is expected that some will emerge as promising therapeutic targets in the future.

DNA methylation is generally associated with gene silencing through the covalent addition of methyl groups to cytosine residues—interfering with the binding of cofactors to DNA—and has been well-described in PAH (reviewed in [173]). Recently, RNA methylation has also been described in PAH [174]. Histone modifications take many forms, the best studied of which are acetylation and methylation of histone tails with subsequent implications for gene regulation [175] and for PAH-relevant phenotypes [176]. Histone methylation has been observed in controlling pathogenic processes in PAH [177]. The discovery of histone acetylation signatures in PAH has catalyzed an intent toward pharmacologic targeting. Bromodomain containing protein 4 (BRD4), a member of the bromodomain and extraterminal domain (BET) family of proteins which bind to acetylated histones, modulates cell cycle progression and inflammation, among others, and has been studied extensively in cancer biology [178]. BRD4 is also upregulated in PAH, and its pharmacologic inhibition ameliorates disease in preclinical models [179]. More recently, inobrodib (CCS1477), a specific bromodomain inhibitor targeting the paralogous histone acetyl transferases p300 and CREB binding protein (CBP) [180], has shown therapeutic efficacy in experimental models [181].

### 4.9 DNA Damage & Senescence

In addition to epigenetic modifications, the accumulation of DNA damage and impaired DNA repair have been described in PAH [182], including in connection with methamphetamine use [183]. In the setting of accumulated damage, as with aging, cells adopt a senescent phenotype with limited proliferative potential yet apoptosis resistance that is accompanied by a pro-inflammatory senescence-associated secretory phenotype (SASP) [184]. An emerging hypothesis positions PAEC senescence as a unifying feature of PAH, based partly on its observation in multiple diverse disease models [132,185]. Interestingly, BRD4 inhibition has been shown to modulate the SASP in cancer cells, potentially contributing to its efficacy in preclinical PH [186]. Additional modulators of senescence, so-called “senolytics” which have been extensively studied in cancer, are ripe for further examination in PAH [184].

### 4.10 Non-Canonical Cell Types & Circulating Bodies

Beyond the well-recognized roles of endothelial, smooth muscle, fibroblast, and immune cells in promoting PAH pathogenesis, there is increasing appreciation for the contributions from non-canonical cell types and circulating bodies in the disease process. It is now recognized that pericytes, subintimal support cells which assist with the maintenance of normal vascular homeostasis (reviewed in [187]), are dysfunctional in PAH and play a role in the pathogenic loss of distal arteriolar beds [188–190]. Recent research has also identified non-canonical functions of well-studied proteins; for example, keratin-1 (KRT1), which is mainly found in hair follicles, has been shown to be regulated by hypoxia and is a negative modulator of PASMC migration and proliferation in experimental PH [191]. Additionally, the role of peripheral nervous system innervation of the pulmonary vasculature is increasingly appreciated [192], and pulmonary artery denervation has shown beneficial signals for the treatment of PAH in uncontrolled studies [193,194]. Stem cell and endothelial progenitor cell biology has been implicated in PAH pathogenesis [195], and endothelial progenitor cell therapy is under clinical study (ClinicalTrials.gov identifier NCT03001414). Mesenchymal stem cells and secreted of circulating microvesicles have displayed therapeutic properties in experimental models. Recent administration of conditioned media from such stem cells resulted in clinical and hemodynamic improvement of severe PAH in a single pediatric patient [196]. Yet, given the extreme pleiotropy of these stem cells and their microvesicle content, identification of the exact causative components of this biology has been challenging. Prior studies have established that miRNAs can be packaged into exosomes to transmit intercellular signals [197], and recent work in our lab demonstrating endocrine delivery of miR-210 during hypoxia in mice with conjoined circulatory systems [198] provides a plausible framework for the effects of circulating microvesicles on pulmonary vascular biology.

### 4.11 Mechanobiology in PAH

Mechanical forces contribute to PAH at the cellular, tissue, and organ levels. The effects of deranged flow patterns are best exemplified in the setting of CHD with systemic-to-pulmonary shunting, although they likely contributed to all subgroups of PAH (reviewed in [199]). Mechanoreceptors on the surface of endothelial cells respond to perturbations in flow [200], with physiologic increases in laminar shear stress leading to activation of NO and PGI2 biosynthetic pathways, downregulation of ET-1, and decreased ROS generation. In this manner, the pulmonary vasculature is able to accommodate increased cardiac output. However, supraphysiologic shear stress and cyclic strain, as seen in the setting of left to right shunting, are accompanied by increases in ET-1, thromboxane A2, ROS production, and pathologic vascular remodeling [201,202].

At the tissue level, vascular stiffness is increased in PAH and correlates with survival [203]. Our group and others have shown that vascular stiffness promotes the activation of that mechanoeffectors YAP and transcriptional co-activator with PDZ-binding motif (TAZ) [204]. The resultant signaling cascades lead to miRNA dysregulation [205], metabolic reprogramming and glutaminolysis [120], downregulation of cyclic oxygenase-2 (COX2) and prostaglandin synthesis [206], and other YAP/TAZ-associated disease mechanisms [207]. In addition to fibrob-last and smooth muscle function, endothelial cell production of collagen may also contribute to pulmonary vascular stiffening [208].

The organ-level response of the RV to increased afterload drives morbidity and mortality in PAH [209]. In the setting of pressure overload, the RV undergoes adaptive concentric hypertrophy which results in decreased wall stress and increased contracticility allowing RV stroke volume to remain “coupled” with its load. At a certain point, cardiac output can only be maintained through maladaptive eccentric hypertrophy (dilation) and tachycardia, eventually leading to RV-PA “uncoupling” with a drop in cardiac output (reviewed in [210]). The detailed molecular underpinnings of RV failure in PAH are incompletely understood, although fibrosis [211], cytoskeletal and sarcomeric remodeling [212], and altered bioenergetics and glutaminolysis [213] are known to play important roles. Interestingly, recent studies have shown that inhibition of IL6 signaling by pharmacologic blockade of its coreceptor, glycoprotein 130 (gp130, also known as IL6ST), attenuates pathologic RV remodeling without impacting the degree of pulmonary vascular remodeling [141,214]. Clinically, morbidity and mortality follow RV dysfunction, which may progress regardless of the use of PVR-lowering therapy [215]. The experimental finding of dissociated vascular and ventricular pathologies adds to growing mechanistic rationale for the development of therapeutics specifically targeting the RV.

### 4.12 Systemic Connections to PAH

It is now clear that a multitude of circulating factors contribute to PAH pathogenesis, including neurohormonal mediators of the renin-angiotensin-aldosterone (reviewed in [216]) and sympathetic nervous systems [217], immune cells and cytokines [163], growth factors, and others. It is also understood that primary disorders of solid organs including the liver, LV, and kidney can result in result in PoPH, Group 2 PH, and Group 5 PH, respectively. The observation that circulating BMP9, a hepatically-synthesized BMPR2 ligand, is decreased in PoPH compared to cirrhosis without PH suggests a direct mechanistic link between PoPH and BMPR2 insufficiency [218,219]. The LV relates to PH in large part due to its interdependence with the RV: in LV dysfunction, elevated filling pressures are experienced as increased afterload by the RV. Meanwhile, RV failure in advanced PAH has significant implications for left ventricular (LV) function, as well—leftward bowing of the inter-ventricular septum and decreased RV stroke volume both necessarily result in decreased LV diastolic filling [220]. Chronic kidney disease (CKD) also coexists frequently with PH [221,222]; while a number of mechanisms have been proposed, including hemodynamic factors observed in cardiorenal syndrome, endothelial dysfunction, and arteriovenous shunting [223], the precise events connecting PH and CKD are unknown. Recently, novel links from pulmonary vascular disease to the gut microbiome [224] and the central nervous system [225] have been proposed, reinforcing the idea of PAH as a systemic disease.

## 5. Presentation & Prognosis

PAH classically presents with nonspecific symptoms of exertional dyspnea and fatigue due to an inadequate increase in cardiac output during activity. Later in the course of the disease, symptoms of RV failure manifest, including leg edema, abdominal distension, early satiety, and near-syncope or syncope [226]. A substantial minority of patients—greater than one third in early registries—will have symptoms of RV failure by the time the diagnosis is established [227]. PAH is associated with significant morbidity and mortality. While limited to 2.8 years prior to the advent of modern therapies [228], median survival is estimated at ~7 years from the time of diagnosis in the current treatment era [229].

Historically, clinical severity and risk of mortality had been categorized primarily by WHO functional class (WHO-FC) [227,230,231], modeled after the New York Heart Association (NYHA) functional classes in heart failure. In the modern era, the synthesis of information across multiple clinical indices and demographics has yielded a more sophisticated algorithm to prognosticate risk of future morbid or mortal events and thus guide therapy. Specifically, once a diagnosis of PAH has been confirmed, an initial risk assessment is performed to gauge prognosis and to guide therapy. Risk stratification is based upon scoring tools derived from PAH registries, including the US-based REVEAL/REVEAL 2.0 [230,232] and the European-based Swedish Pulmonary Arterial Hypertension Register (SPAHR) [233], Comparative, Prospective Registry of Newly Initiated Therapies for Pulmonary Hypertension (COMPERA) [234], and French Pulmonary Hypertension Registry (FPHR) [235]. While varying in their precise formulations, all tools use a combination of clinical, functional, exercise, hemodynamic, and biochemical inputs to assign a risk category (low, intermediate, or high) and inform initial management strategies [236]. Importantly, current guidelines endorse the use of serial risk assessments at 3–6 months with a goal of maintaining or achieving a low-risk profile through escalation of therapy [102].

## 6. In Pursuit of Early Diagnosis

Given the apparent efficacy of early therapy at reducing mortality [229,237], a major focus of PAH management has been on the early diagnosis of disease [238]. The initial symptoms of PAH are nonspecific and require a degree of clinical suspicion in order to pursue a thorough diagnostic evaluation. This workup is burdensome and typically begins with transthoracic echocardiography (TTE). When TTE shows features consistent with pulmonary hypertension, or if uncertainty remains, an invasive hemodynamic assessment with right heart catheterization should be performed. If right-sided hemodynamics are consistent with a diagnosis of PH, additional imaging and serologic studies must be performed to rule out more common causes of PH and establish a diagnosis of PAH [102].

In the REVEAL registry, more than 20% of patients experienced a delay of greater than 2 years between the onset of symptoms and the diagnosis of PAH [239]. As PAH is a progressive disease and more advanced disease is associated with poor outcomes, it is unsurprising that early diagnosis and treatment of PAH is critical to improving survival [237]. Detection of early or pre-symptomatic PAH is difficult, due in part to the very nature of the disease [238]. Recent studies have found that hemodynamic values of mPAP [240] and PVR [241] previously considered as “borderline” in fact portend worse clinical outcomes, prompting alterations to the hemodynamic definitions of clinically significant disease [2]. Even in familial PAH, the low penetrance of disease-associated mutations [242] means that genetic testing alone is insufficient to identify individuals who will develop clinical disease. In addition, physiologic adaptation of the right ventricle to increased afterload may delay clinical symptoms until disease is already present. Furthermore, intrinsic reserves constituting greater than 60% of the pulmonary vascular cross-sectional area allow for normal resting hemodynamics even when pathogenic remodeling is well underway [243].

In the face of these challenges, the development of improved screening and risk-stratification tools has become an area of intense interest. One area that has received prolonged attention but has yet to see widespread adoption is invasive cardiopulmonary exercise testing (iCPET) [244]. In theory, iCPET can serve as a “stress test” for the pulmonary circulation and unmask latent PAH: with the obliteration of pulmonary vascular beds and reduction in vascular distensibility, early PAH would be expected to be accompanied by a disproportionate rise in mPAP during exercise. However, the lack of clear data on what constitutes an abnormally elevated mPAP during exercise, and the difficulty in distinguishing between pre- and post-capillary causes of such elevations, have made it challenging to include exercise PH in current guidelines [2]. In addition, technical requirements and limited access are hurdles to the widespread adoption of iCPET. Nonetheless, interest in iCPET as a screening modality has continued, with some investigators advocating its use in the identification of affected carriers in familial forms of disease [245] or those with known risk factors of PAH.

### 6.1 Novel Imaging Platforms

While invasive hemodynamic measurements remain the gold standard in assessing the presence and severity of PAH [2], they ultimately reveal phenotypic rather than histopathological insights. As a result, they are a lagging indicator of disease progression and, as mentioned, remain normal until severe pathologic alterations have already taken place. Similarly, widely used imaging modalities such as echocardiography and cardiac magnetic resonance imaging (MRI) are useful in assessing and monitoring phenotypic consequences—including elevations in pulmonary artery pressures and declines in right ventricular systolic function—of pathologic events [246]. 4D flow MRI is a recent advancement which combines three-dimensional spatial encoding with three-directional velocities to allow for improved hemodynamic assessment, although its application to PAH is in its early stages [247]. Magnetic resonance spectroscopy (MRS) is an older technology with the ability to provide add molecular quantitation to traditional imaging data, and its experimental use to quantify metabolites in the failing RV suggests that it may have clinical application, as well [248,249]. More recently, novel molecular imaging modalities have been developed which, if translatable to the clinical realm, may be able to identify PAH before disease is clinically evident. ^129^Xe MRI is an emerging pulmonary imaging technique which utilizes the stable xenon isotope ^129^Xe to generate three dimensional maps of lung uptake, interstitial diffusion, and erythrocyte transfer of gaseous or soluble ^129^Xe [250]. Applied to animal models and two patients with PAH [251,252], ^129^Xe MRI revealed a signature impairment in erythrocyte transfer that was distinct from other studied lung pathologies and preceded the onset of severe disease in rodents. A second emerging technology, positron emission tomography (PET) imaging utilizing a macrophage-targeting tracer identified rodent disease prior to hemodynamic derangements and was able to distinguish PAH from PH-LHD in a small cohort of human subjects [253]. Such molecular imaging techniques have the potential to fundamentally alter the diagnostic evaluation of PAH, shifting the process from procedural assessments of late phenotypic sequelae to noninvasive measurements of early pathologic derangements.

### 6.2 Biomarkers

A perhaps simpler means of disease detection would be to through the use of a diagnostic blood test. Although biomarkers have seen robust interest, there has been limited success in their application to PAH [254]. B-type natriuretic peptide (BNP) is perhaps the most widely-used biomarker in PAH, a preformed peptide release from the ventricle during periods of increased wall tension that correlates with hemodynamic derangements [255], RV systolic function [256], and mortality [257]. However, it does not distinguish between right- and left-sided heart disease; even after controlling specificity in a high-risk scleroderma population, NT-proBNP performed poorly (56% sensitivity) in the detection of early disease [258]. Therefore, the identification of circulating factors that are both specific to PAH and sensitive to early pathology is essential to the development of clinically useful biomarkers.

In order to improve biomarker specificity, investigators have examined mechanistic biomarkers that may better reflect underlying pathologic processes in retrospective analyses. In one such recent study, the novel biomarker NEDD9 was found to be increased in PAH [208]. In another small study, our group proposed Signal peptide, CUB domain and EGF like domain containing 1 (SCUBE1) as a mechanistic biomarker of PAH based on its differential expression in induced pluripotent stem cell endothelial cells (iPSC-Ecs) derived from affected and carrier *BMPR2* mutant heterozygotes. Plasma SCUBE1 levels were able to distinguish PAH from controls and the other more common WSPH Groups 2 and 3 PH [259]. In addition to peptides, microRNAs are known to mediate crucial pathogenic processes in PAH, and circulating disease-relevant microRNAs have been proposed as biomarkers of early disease (reviewed in [170]). While these assays are far from clinical deployment, it is clear that similar mechanistic approaches will be essential to bringing a useful biomarker into clinical practice.

Of course, PAH, while hemodynamically defined as a single disease, can arise from several distinct etiologies. Nikolic and colleagues recently showed that circulating levels BMP9, a ligand for BMPR2 synthesized in the liver, are significantly reduced in PoPH but not in other forms of PAH [219]. This heterogeneity within various subtypes of PAH suggests that multiple biomarkers or molecular panels may be necessary to provide early and accurate diagnoses.

## 7. Current & Future Therapies

Pulmonary vasodilators, which predate our current understanding of disease mechanisms, form the backbone of pharmacotherapy in PH. These drugs fall into three categories depending on the targeted pathway—prostanoids, nitric oxide potentiators (phosphodiesterase 5 [PDE5] inhibitors and soluble guanylate cyclase [sGC] activators), and endothelin receptor antagonists (ERA)—and have been extensively reviewed previously [13]. Additionally, high-dose calcium channel blockers (CCBs) are indicated in a small subset of PAH patients who respond to invasive vasoreactivity testing [102]. Current recommendations indicate that, in low and intermediate-risk patients, initial combination therapy with a PDE5 inhibitor and ERA is appropriate. Meanwhile, high-risk patients should be started on combination therapy which includes an intravenous prostanoid. On sequential assessment, patients at low risk may be continued on their current regimens, while those at intermediate or high risk should advance to triple combination therapy including a PDE5 inhibitor, ERA, and intravenous prostanoid [236]. The era of vasodilator therapy has been accompanied by improvements in quality and quantity of life [229], although vasodilators do not reverse the pathological features of PAH. When medical therapy fails, lung or heart-lung transplantation is the only option [260], highlighting the need for effective and targeted therapeutics.

### 7.1 Drugs Targeting BMPR2 Signaling

With greater understanding of disease mechanisms, drug-development efforts have shifted from nonspecific vasodilators to targeted therapeutics. Chief among these targeting strategies are drugs that aim to restore balance between BMPR2 signaling—which is diminished in hereditary and other forms of PAH—and TGF*β* signaling, which is increased. Sotatercept, initially developed to treat osteoporosis, is a fusion protein consisting of the extracellular domain of human activin receptor type IIA and the Fc domain of IgG1 which serves as a ligand trap for members of the TGF-*β* superfamily thereby decreasing pro-growth SMAD2/3 signaling to restore balance with the growth-inhibiting SMAD1/5/9 signaling diminished by BMPR2 insufficiency [48]. In a recent randomized controlled trial, sotatercept treatment resulted in a significant decrease in pulmonary vascular resistance among patients on maximum tolerated background PAH therapy [261]. Alternatively, the augmentation of BMPR2 can also rebalance the BMPR2/TGF*β* scale; the BMPR2 ligand BMP9 has been proposed as a means of restoring balanced SMAD signaling in the pulmonary vasculature and has shown efficacy at reversing PAH in preclinical studies [262]. Similarly, the immunosuppressive drug tacrolimus (FK506) used in transplant recipients was identified from a screen of more than 3500 compounds as harboring potent BMPR2 agonism [263]. Tacrolimus prevented and reversed pulmonary hypertension in multiple rodent disease models, and clinical trials are planned [264].

### 7.2 Repurposing of Cancer Therapies

As illustrated by the application of tacrolimus to PAH, repurposing of existing drugs to the treatment of PAH has emerged as a strategy to overcome the costs of *de novo* drug development and the inherent difficulty of conducting clinical trials in rare diseases [265]. Cancer therapies have attracted significant interest in PAH given the substantial mechanistic overlap between cancer and PH [266]. As with cancer, tyrosine kinase receptors (TKRs) play crucial roles in transmitting mitogenic signals to the pulmonary arterial smooth muscle resulting in pathogenic hypertrophy and hyperplasia [267]. This knowledge spurred interest in the study of the tyrosine kinase inhibitor (TKI) imatinib, a partially selective inhibitor of the platelet-derived growth factor receptor approved for the treatment of chronic myelogenous leukemia, for the treatment of PAH. While imatinib was efficacious at improving symptoms and functional class—as well as reversing disease in preclinical models—the high rate of severe adverse events, notably subdural hematomas, precluded its clinical use [267,268]. In order to minimize off-target effects, inhaled TKIs have been developed, including aerosolized imatinib (AV-101) and seralutinib (GB002) which are currently in clinical trials for the treatment of patients with PAH on background vasodilator therapy (ClinicalTrials.gov identifiers NCT05036135, NCT04816604) [269,270]. Paradoxically, the TKI dasatinib—and potentially others—has been linked to the development of PAH [271,272]. While the precise mechanisms of these divergent effects are unclear, they may be a consequence of variable TKR specificity profiles, including Src inhibition, as well as other mechanisms [273].

The case of TKIs shows the challenges of predicting cumulative drug effects based on mechanism alone. One strategy to address this concern is to infer net effects based on predictive algorithms. Our group recently analyzed transcriptomic differential dependency networks of a library of cancer drugs [274] to identify compounds leading to the rewiring of PH gene clusters. This approach led to the identification of a bromodomain-containing protein BRD2/4 inhibitor and a piperlongumine-like GSTP1 inhibitor, both of which ameliorated experimental PH [275]. Correspondingly, the BRD4 inhibitor JQ1 has previously been shown to reverse experimental PH in rodent models [179], and the BRD4 inhibitor apabetalone (RVX208) is currently under Phase 2 clinical investigation in PAH (ClinicalTrials.gov identifier NCT04915300) [276,277].

Several additional cancer therapeutics have garnered interest in PAH, including anastrazole and tamoxifen targeting estrogen signaling [89,278]; palbociclib-mediated cyclin-dependent kinase 4/9 (CDK4/9) inhibition [279]; and modulation DNA damage/repair with the poly-ADP ri-bose polymerase inhibitor olaparib [182], highlighting the overlapping pathophenotypes between PAH and cancer as well as the hope that these drugs can be successfully translated to the clinical management of pulmonary vascular disease.

### 7.3 Drugs Targeting Metabolic Dysregulation

Similar to cancer, metabolic reprogramming from oxidative phosphorylation to glycolysis under aerobic conditions—known as the Warburg effect—is a core feature of PAH associated with aberrant activation of proliferative pathways and adverse RV remodeling [106]. In addition to the aforementioned trial of DCA in PAH, other metabolic drugs are under investigation. As discussed earlier, cells must maintain adequate biomass to sustain proliferation through anaplerosis. ECM stiffening characteristic of PAH stimulates glutaminolytic generation of TCA carbon intermediates through the activation of a YAP-GLS1 molecular axis to sustain pulmonary vascular cell proliferation through YAP1-dependent upregulation of GLS1 [120,207]. Both the YAP inhibitor verteporfin, used in the treatment of macular degeneration [280], and GLS1 inhibitor CB-839 ameliorated cellular proliferation and PH in multiple rodent and primate disease models [120]. Given the ubiquitous expression of YAP1 and GLS1 and in order to minimize systemic toxicities, an inhaled delivery system was developed that a showed a synergistic benefit of combined verteporfin and CB-839 therapy in the treatment of experimental PH [118], establishing these drugs and drug targets, singly or in combination, as promising candidates for further development.

The distressed right ventricle also undergoes metabolic rewiring in advanced PAH whereby the normal balance between glucose and fatty acid utilization, established through substrate competition in a process known as the Randle Cycle, is disrupted in favor of increased fatty acid oxidation [213,281]. By inhibiting fatty acid oxidation, it has been shown that fatty acid oxidase (FAO) inhibitors can shift metabolic substrates toward glucose oxidation and thereby improve right ventricular function [106,282]. Ranolazine and trimetazidine, two FAO inhibitors used clinically to treat refractory angina pectoris [283], increased RV cardiomyocyte glucose oxidation, reversed RV hypertrophy, and improved exercise capacity in a PA-banding model of RV pressure-overload failure [213]. In independent small human pilot studies, ranolazine was found to improve various clinical aspects of right ventricular function and size in PAH [284,285]. Trimetazidine is likewise the subject of active clinical trials investigating its impact on RV function and metabolism in PAH.

The repurposing of medications used in diabetes mellitus, specifically the PPAR*γ* agonist thiazolidinediones (TZDs) and the AMP-activated protein kinase (AMPK) stimulator metformin, has also been of interest in PAH, in part based on findings that insulin resistance is common in the disease [133,135,286]. However, the beneficial mechanisms of these medications in PAH are believed to extend beyond their antihyperglycemic effects. PPAR*γ* is a transcriptional regulator of key enzymes involving glucose and fatty acid utilization which is suppressed in experimental PH and linked to BMPR2 signaling [137,287]. Pharmacologic activation of PPAR*γ* with the TZDs rosiglitazone or pioglitazone has been consistently shown to prevent and reverse PH in preclinical models [119,135,136]. In light of the beneficial effects of FAO inhibitors on RV performance, it is counterintuitive that TZDs have shown a beneficial effect on RV function attributed to *increased* fatty acid utilization [136], perhaps best explained by the restoration of glucose/fatty acid homeostasis rather than an intrinsic preference for a particular fuel source. Despite strong evidence of benefit in animal models, concerns about the cardiac risk profile of TZDs—namely, their association with heart failure exacerbations [288,289]—have thus far prevented their advancement to clinical trials in PAH. Metformin, meanwhile, a well-tolerated first-line anti-diabetic agent, ameliorates vascular cell proliferation and RV dysfunction in multiple animal models as well as in a small human cohort [290,291]. Metformin has also shown to be therapeutic in preclinical models of Group 2 PH, and debate persists over whether the observed benefits of the drug are limited to metabolic syndrome-associated diastolic heart failure or truly extend to Group 1 PAH [292]. In fact, momentum appears to be shifting away from the study of metformin and TZDs. However, given the preclinical successes of the newer antihyperglycemic agents of the sodium glucose cotransporter-2 (SGLT2) inhibitor [293] and glucagon-like peptide-1 (GLP1) receptor agonist [294] on the treatment of experimental PH and in human trials of heart failure with PH in general, it is expected that these drugs will soon advance to clinical trials in PAH.

### 7.4 Drugs Targeting Inflammation & Immunity

As discussed, inflammatory factors and immune mediators are tightly linked to the signature pathogenic changes in PAH [163]. They have received attention as potential therapeutic targets but with less robust results. IL-6, a central inflammatory cytokine produced by vascular and non-vascular cells, is quantitatively associated with PAH outcomes [295], and forced overexpression of its receptor IL6R causes vascular remodeling in animal models of PH [150]. Tocilizumab, a humanized monoclonal antibody targeting IL6R and approved for use in certain diseases such as cytokine release syndrome, has shown efficacy at reversing disease pathology in preclinical models. However, human data have so far been less compelling [296], with a small 6-month phase 2 study showing a decrease in serum inflammatory markers but no change in pulmonary vascular resistance or functional outcomes [151]. Interestingly, a modest reduction in PVR was noted in four of six patients with CTD-PAH which, interpreted cautiously, may suggest that particular subsets will respond favorably to tocilizumab therapy. In a similar fashion, a small randomized-controlled pilot study of B-cell depletion therapy in SSc-PAH produced mixed results, with low levels of rheumatoid factor (RF), IL-12, and IL-17 predictive of improvements in 6-minute walk distance after rituximab therapy [297]. Collectively, these results indicate that enhanced strategies to align patients with individualized anti-inflammatory regimens may improve therapeutic responses.

## 8. Precision Medicine (Fig. 2)

PAH is a heterogeneous disorder with a multitude of causes as outlined in this review. It is already well-established that subsets of PAH patients—notably those with PVOD/PCH [10]—may not respond favorably to existing vasodilator therapies. As the pharmacologic arma mentarium of PAH expands, it is unlikely that all patients will derive equal benefit from targeted therapies. For example, it has already been suggested that individuals with CTD-PAH may be more likely to benefit from anti-inflammatory biologics [151], while polymorphisms in certain endothelin-related genes may predict the clinical response to ERAs [298]. Hence, matching the appropriate therapy to the proper patient will become paramount, particularly if more drugs are to be tested appropriately in the limited global number of PAH patients available for recruitment. The National Research Council defines precision medicine as the “tailoring of medical treatment to the individual characteristics of each patient” [299]. Given the diversity of pathologic insults resulting in PAH, it is reasonable to expect that individualized care will yield benefits in patient outcomes.

One could consider an early observation in PAH therapy as an example of precision medicine before it was known as such. It has long been recognized that calcium channel blockers (CCBs) cause an acute vasodilator response in a small subset (less than 10%) of patients with IPAH [300]. In clinical studies, responders have been observed in idiopathic, heritable, and anorexigen-induced PAH [301,302] and are identified by an acute vasodilator response to nitric oxide, epoprostenol, or, less commonly, adenosine during invasive hemodynamic testing [302]. When treated with long-term CCB therapy, such patients have markedly improved survival compared to non-responders [302,303]. More recently, transcriptomic signatures in peripheral blood samples have shown the ability to differentiate vasoreactive and non-vasoreactive patients with high sensitivity and specificity [304], suggesting a unique molecular phenotype of CCB responders. The distinct clinical and molecular profile of CCB responders led to their inclusion as a separate subset of PAH in the most recent clinical classification guidelines [2]. One goal of precision medicine is to identify the contours of additional subgroups so that they may be targeted with specific therapies.

Early attempts to apply deep omics-level phenotyping to PAH have already begun, including genomic [37], transcriptomic [305], proteomic [306], metabolic [307] and immune [164] profiling of PAH subjects. As a proof of concept, Sweatt and colleagues [164] recently utilized a machine learning approach to identify 4 immune clusters in PAH based on cross-sectional levels of 48 circulating cytokines, chemokines, and growth factors. Despite the inclusion of numerous PAH subgroups, the identified clusters did not correlate with clinical classifications but were strongly predictive of survival. Hence, the these clusters may represent a surrogate of disease severity rather than distinct molecular phenotypes, a possibility that will be addressed by future studies with longitudinal data. Such an effort is currently underway—the Pulmonary Vascular Disease Phenomics Program (PVDOMICS)—that seeks to redefine PH subgroups in place since 1998 based on clusters identified through deep phenotyping [308].

With vast quantities of population and patient-specific information spanning the molecular, genomic, radio-graphic, demographic, and clinical realms, novel computational methods employing multiscale modeling and machine learning will be required to integrate these data into clinically meaningful tools. If employed successfully, such algorithms have the potential to provide improved diagnostic and risk assessment platforms, inform research directions and drug development, and guide patients toward tailored therapies and clinical trials.

## 9. Conclusions

The past 30 years have brought multiple gains to the management and prognosis of PAH. However, the clinical application of fundamental discoveries and technological advances developed in this time frame promises to accelerate this trajectory. Pulmonary hypertension is a field where basic science and clinical care are rapidly evolving together, and it will benefit our patients to have clinicians who are well-versed in the two. With improved diagnostic capabilities and expanded treatment options tailored to well-defined molecular phenotypes, the future of precision PAH management is promising.

## Figures and Tables

**Fig. 1.**
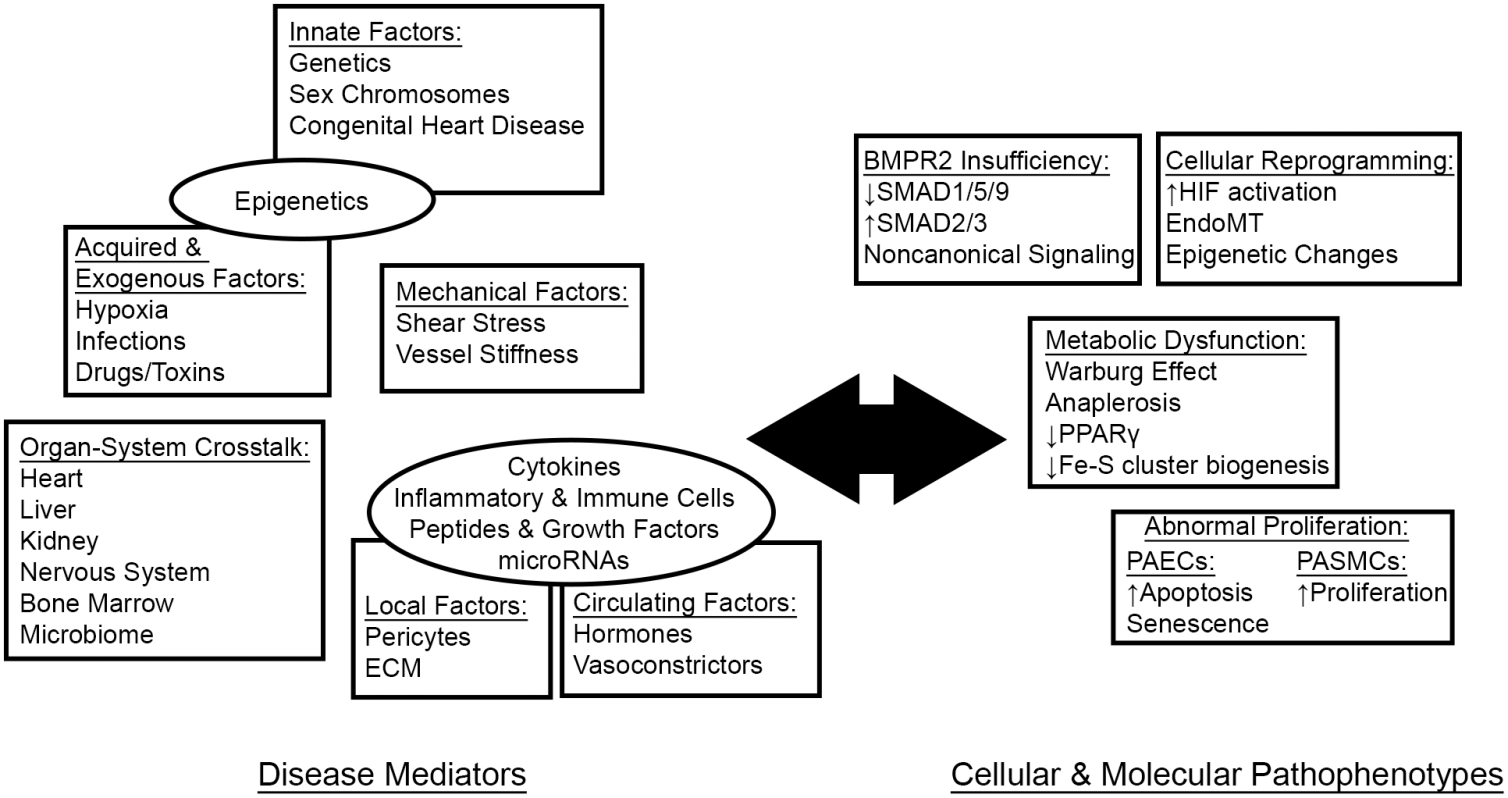
PAH as a systemic disease. A growing body of literature reveals the interplay between systemic disease mediators and cellular pathophenotypes in driving pathologic vascular pathology in PAH. ECM, extracellular matrix.

**Fig. 2.**
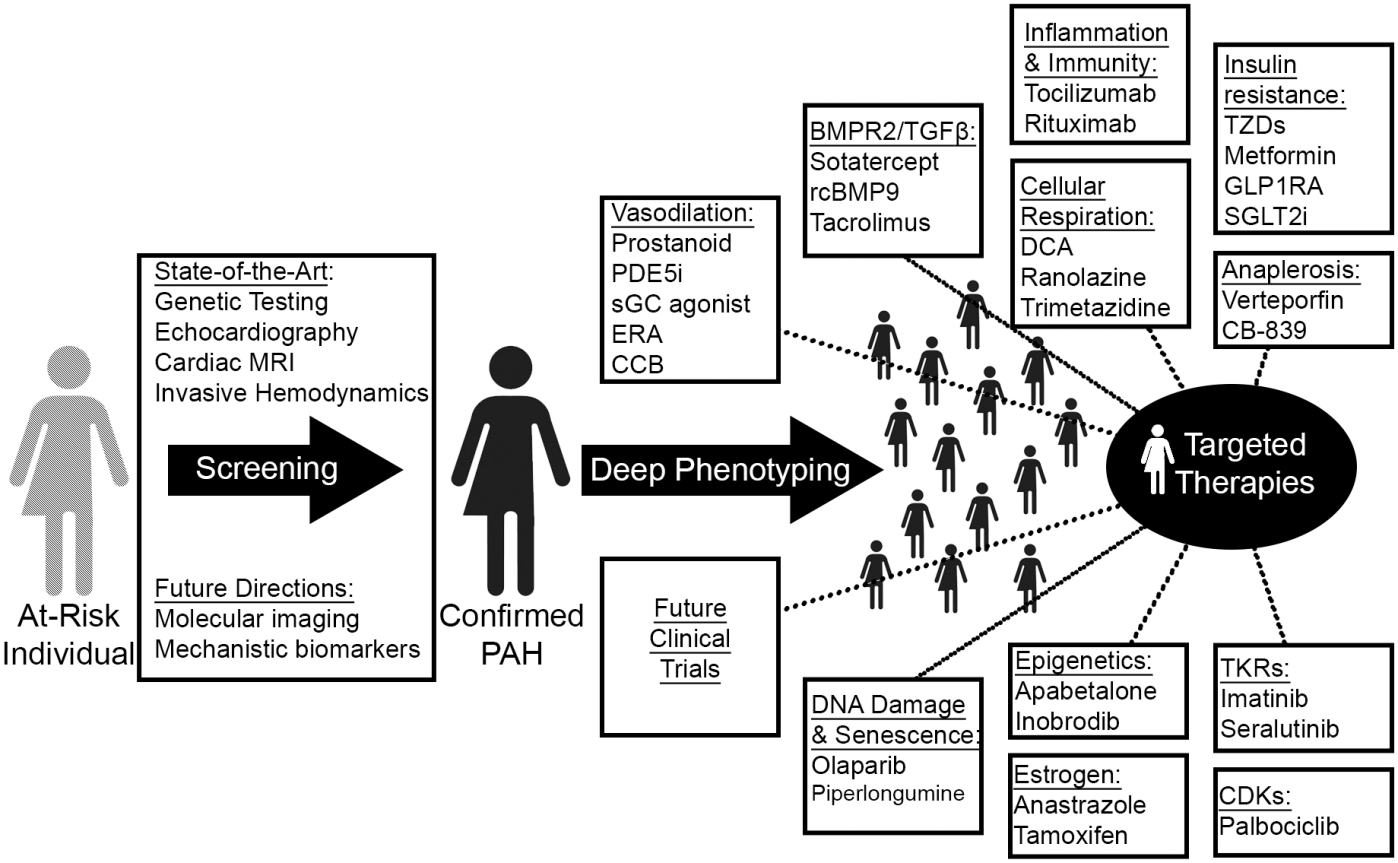
Precision medicine and novel treatment paradigms in PAH. Guided by improved diagnostic technologies and omics-level deep phenotyping, therapeutic targeting of novel PAH-relevant processes will match a potentially new and molecularly-guided catalog of disease clusters to tailored regimens. MRI, magnetic resonance imaging; PDE5i, phosphodiesterase 5 inhibitor; sGC, soluble guanylate cyclase; ERA, endothelin receptor antagonist; CCB, calcium channel blocker; rcBMP9, recombinant bone morphogenic protein 9; GLP1RA, glucagon-like peptide 1 receptor agonist; SGLT2i, sodium-glucose cotransporter-2 inhibitor; TKRs, tyrosine kinase receptors; CDKs, cyclin-dependent kinases.
